# Retrospective Analysis of Atypical Chest Pain Presentations in Older Adults and Their Association With Missed Acute Coronary Syndrome Diagnosis in the Emergency Department: National Hospital Ambulatory Medical Care Survey (NHAMCS)-Based Study

**DOI:** 10.7759/cureus.88496

**Published:** 2025-07-22

**Authors:** Lambi Jervis Somo, Okelue E Okobi, Amarachi Asomugha-Okwara, Kimberly Osias, Peace O Mordi, Edidiong Enyeneokpon, Paul O Etakewen, Cynthia Okoro

**Affiliations:** 1 Internal Medicine, Mbingo Baptist Hospital/Christian Internal Medicine Specialization, Bamenda, CMR; 2 Family Medicine, Larkin Community Hospital Palm Springs Campus, Miami, USA; 3 School of Epidemiology and Public Health, Faculty of Medicine, University of Ottawa, Ottawa, CAN; 4 Family Medicine, Université Notre Dame D'Haiti, Port-au-Prince, HTI; 5 Family Medicine, Benton Medical Clinic, Kitchener, CAN; 6 Acute Medicine, Pilgrim Hospital, Boston, GBR; 7 Emergency Medicine, Milton Keynes University Hospital, Milton Keynes, GBR; 8 Family Medicine, Richmond Gabriel University, Arnos Vale, VCT

**Keywords:** acute coronary syndrome, atypical chest pain, emergency department, missed diagnosis, older adults

## Abstract

Background

Atypical symptom presentations of Acute Coronary Syndrome (ACS) are common in older adults and may contribute to diagnostic delays or missed recognition in emergency departments (EDs). National-level data examining this relationship remains limited.

Objective

To evaluate whether atypical chest pain presentations are associated with reduced likelihood of ACS diagnosis among U.S. adults aged 65 years and older during ED visits.

Methods

We conducted a retrospective cross-sectional study using data from the National Hospital Ambulatory Medical Care Survey (NHAMCS) from 2014 to 2020. ED visits by adults aged ≥65 years were analyzed. Atypical presentations were defined using Reason for Visit (RFV) codes for symptoms such as weakness, dyspnea, dizziness, nausea, syncope, and abdominal pain. The primary outcome was an ED diagnosis of ACS based on ICD-9-CM codes. Multivariable logistic regression was used to assess associations.

Results

Among 2,470 eligible ED visits, only 15 (0.6%) were diagnosed with ACS. Of those, 7 (46.7%) presented with atypical symptoms. Atypical presentation was not significantly associated with ACS diagnosis (OR: 0.90; 95% CI: 0.32-2.49; p = 0.83). No significant associations were found with age, sex, race/ethnicity, or ED disposition. The variable "admitted to hospital from ED" was excluded due to collinearity.

Conclusion

Nearly half of older adults diagnosed with ACS presented atypically, yet atypical presentation was not significantly associated with missed ACS diagnosis in the ED. Given the limitations of administrative data and low ACS event rates, future research using richer clinical datasets and follow-up outcomes is needed to better understand diagnostic gaps in this high-risk population.

## Introduction

Recently, in the United States, chest pain has been among the common causes of adult visits to emergency departments (EDs). In older people, it is also a possible indication of severe underlying diseases such as acute coronary syndrome (ACS), comprising unstable angina and myocardial infarction [1]. ACS management is essential through early diagnosis and intervention among the elderly. The symptoms, however, are usually not typical in this age group [2]. This poses a problem of diagnosis that may lead to late or even incorrect diagnosis [3].

In contrast to patients who are younger and who mention classic chest pain, central, crushing, and radiating, older adults tend to complain of nonspecific or vague symptoms [4, 5]. This could be fatigue, shortness of breath, dizziness, nausea, or general body discomfort. Gastrointestinal problems, such as indigestion or abdominal pains, are the only indicators in some cases [6]. The symptoms may not be found in the textbook, thus making many clinicians first reject the prospect of a cardiac origin, but this may be very harmful [7]. Older people with misdiagnosed or delayed diagnosis of ACS had worse outcomes, such as higher deaths, heart failures, and even longer stays [7, 8].

Several systemic and physiological factors explain this diagnostic gap. Old-age individuals tend to have more diseases, are prescribed medication, and show different or dulled physiological reactions [6, 9, 10]. Such characteristics have the potential to conceal the classic manifestations of myocardial ischemia [2, 4, 11]. Besides, emergency physicians, in general, have to deal with the problem of overcrowding in EDs and time limits, which adds potential value to the reliance on pattern recognition that gives a preference to younger patients with classic presentations [7, 12].

Unusual symptoms are more likely to be missed in women and racial minorities, both of whom are overrepresented among the older population [4, 6, 13]. These differences may enlarge the gap in diagnosing and timely diagnosis of ACS [14]. Although the guidelines admit unusual presentations, they tend not to provide age-related guidelines. Consequently, old patients can fail to undergo prompt electrocardiograms (ECGs) and cardiac biomarker tests, which are paramount in quick checkups on ACS [15].

The development of ACS diagnosis with high-sensitivity troponins has enhanced its detection. Nevertheless, presenting symptoms are critical as the first stage of triaging and prioritization [2, 7, 16]. When the symptoms do not match the mold, patients are treated conservatively or discharged earlier [10, 17]. This highlights why it is essential to establish how the existence of atypical chest pain influences the results of diagnosis in the real-life ED environment [18].

The study will utilize National Hospital Ambulatory Medical Care Survey (NHAMCS) data. The evaluation of this data can provide some information about the trends, including the likelihood of older adults with atypical chest pain being misdiagnosed or underdiagnosed with ACS [19]. Although previous studies examined ACS in the general population or merely identified rare symptoms among a small sample of potential cases, little has been done to explore this problem nationally [20]. The research objective is to examine the relationship between atypical chest pain symptoms in U.S. adults aged 65 years and older and the likelihood of missed or delayed Acute Coronary Syndrome (ACS) diagnoses during ED visits, using NHAMCS data [21].

## Materials and methods

Study design and data sources

This study employed a retrospective, cross-sectional design using publicly available data from the National Hospital Ambulatory Medical Care Survey (NHAMCS), collected by the National Center for Health Statistics (NCHS) [21]. NHAMCS provides nationally representative data on visits to emergency departments (EDs) across the United States, including patient demographics, presenting symptoms, diagnoses, procedures, and outcomes. The survey uses a multistage probability sampling design and applies survey weights to allow national estimates. For this study, data from 2014 to 2018 were used, the most recent years available with consistent variables required for the analysis.

Study population

The analysis was restricted to ED visits by adults aged 65 years and older. Visits with missing data for key variables, including age, diagnosis codes, or symptom fields, were excluded. Only cases with complete information relevant to ACS identification and symptom classification were retained for analysis.

Variable definitions

The primary exposure variable was symptom presentation, derived from the Reason for Visit (RFV) fields (RFV1, RFV2, RFV3). A typical presentation was defined by the presence of RFV code 10501, indicating chest pain. Atypical presentations were identified using RFV codes corresponding to non-classical symptoms associated with Acute Coronary Syndrome (ACS), including shortness of breath (14150), dizziness or vertigo (12250), nausea (15250), vomiting (15300), syncope or fainting (10300), general weakness (10200), and abdominal pain (15451). In cases where both typical and atypical symptoms were present, patients were categorized as having a typical presentation. This approach reflects clinical practice, where the presence of chest pain typically prompts early consideration of ACS.

The primary outcome was the presence or absence of an ACS diagnosis made during the ED visit, as determined by ICD-9-CM diagnosis codes recorded in the first three ED diagnosis fields (DIAG1, DIAG2, DIAG3). A binary outcome variable (acs_diag_ed) was created to indicate whether an ACS diagnosis was made. Additionally, several covariates were included in the multivariable analysis based on their clinical relevance and supporting literature. These included age (continuous, in years), sex (male or female), and race/ethnicity, categorized from the RACERETH variable into four groups: non-Hispanic White, non-Hispanic Black, Hispanic, and non-Hispanic Other. Emergency department disposition was also included, based on the ADISP (Final disposition from ED) variable, and categorized as discharge, transfer to another facility, or return/transfer to a nursing home. Although hospital admission (ADMITHOS) was initially considered, it was excluded from the final regression model due to perfect collinearity, as all patients diagnosed with ACS were admitted.

Statistical analysis

Descriptive statistics were used to summarize patient characteristics by ACS diagnosis status. Categorical variables were presented as frequencies and percentages, while continuous variables were summarized using means and standard deviations. Group differences were assessed using chi-square tests for categorical variables and t-tests for continuous variables. Also, a multivariable logistic regression model was constructed to examine the association between atypical presentation and the likelihood of ACS diagnosis in the ED. The model was adjusted for age, sex, race/ethnicity, and ED disposition. The final model excluded “admitted to hospital from ED” due to collinearity (all ACS-diagnosed patients were admitted). Results were reported as adjusted odds ratios (ORs) with 95% confidence intervals (CIs). Statistical significance was set at p < 0.05. All statistical analyses were conducted using Stata version 18 and accounted for the complex survey design of NHAMCS by applying the appropriate survey weights to produce nationally representative estimate (StataCorp, College Station, TX, USA) [22].

Ethical considerations

This study used de-identified, publicly available data from the NHAMCS database. As such, it was considered non-human subjects research and did not require institutional ethics board approval.

## Results

A total of 2,470 emergency department (ED) visits among adults aged 65 years and older were analyzed from the NHAMCS 2014-2020 dataset. Among these, only 15 patients (0.61%) received an ED diagnosis of Acute Coronary Syndrome (ACS). Table 1 below presents a comparison of demographic and clinical characteristics between patients who were diagnosed with ACS in the ED and those who were not.

**Table 1:** Comparison of Patient Characteristics by ACS Diagnosis in the Emergency Department Table 1 displays the distribution of patient demographics and presentation characteristics stratified by whether an ACS diagnosis was made during the ED visit. Continuous variables are summarized using means and standard deviations, and categorical variables are presented as frequencies and percentages. P-values were calculated using t-tests for continuous variables and chi-square tests for categorical variables. Chi-square testing did not apply to hospital admission status due to no variability in the outcome group, denoted by N/A. NA: Not Analyzed Asterisks indicate statistical significance: * p < 0.05, ** p < 0.01, *** p < 0.001

Variable	ACS diagnosed in ED (n=15)	No ACS diagnosed in ED (n=2455)	T-test	Chi-square	P-value
Age (in years)	80.27 ± 8.55	77.83 ± 8.34	-1.10	NA	0.27
A typical symptom presentation: Yes	7(46.67%)	1234(50.26%)	NA	0.08	0.78
No	8(53.33%)	1221(49.74%)	NA		
Sex: Male	7(6.4%)	1083(99.4%)	NA	0.04	0.84
Female	8(0.6%)	1372(99.4%)	NA		
Race/Ethnicity: Non-Hispanic White	12(0.6%)	1888(99.4%)	NA	0.75	0.86
Non-Hispanic Black	2(0.6%)	304(99.4%)	NA		
Hispanic	1(0.7%)	147(99.3%)	NA		
Non-Hispanic Other	0	116(100%)	NA		
Admitted to hospital from ED: Yes	15(0.6%)	2455(99.4%)	NA	N/A	N/A

The mean age of patients diagnosed with ACS in the ED was 80.27 years (SD: 8.55), compared to 77.83 years (SD: 8.34) among those without an ACS diagnosis; this difference was not statistically significant (t = -1.10, p = 0.27). Atypical symptom presentation was present in 7 (46.67%) of patients with ACS and 1234 (50.26% ) of those without, with no statistically significant difference (χ² = 0.08, p = 0.78). Similarly, there were no significant differences in sex distribution (χ² = 0.04, p = 0.84), with males representing 7 (6.4%) of the ACS group and 1083 (99.4%) of the non-ACS group. Still, for race and ethnicity, 12 (0.6%) ACS cases were non-Hispanic White, 2 (0.6%) were non-Hispanic Black, and 1 (0.7%) were Hispanic. No ACS cases were observed among non-Hispanic Other individuals. Among those without ACS, 1,888 (99.4%) were non-Hispanic White, 304 (99.4%) were non-Hispanic Black, 147 (99.3%) were Hispanic, and 116 (100%) were non-Hispanic Other. Differences across race/ethnicity were not statistically significant (χ² = 0.75, p = 0.86). Notably, all patients diagnosed with ACS in the ED were admitted to the hospital (100%), which made statistical testing for admission status not meaningful due to the absence of a comparison group (χ² = N/A).

Multivariable logistic regression

To identify factors independently associated with an ACS diagnosis made in the emergency department, a multivariable logistic regression model was constructed as shown in Table 2 below. The outcome variable was ACS diagnosis in the ED (yes/no). Predictor variables included atypical symptom presentation, age, sex, race/ethnicity, and ED disposition.

**Table 2:** Multivariable Logistic Regression: Factors Associated with ACS Diagnosis in the Emergency Department Table 2 presents adjusted odds ratios from a multivariable logistic regression assessing factors associated with ACS diagnosis made during the emergency department visit. The model included variables selected a priori based on clinical relevance. Non-Hispanic White and standard discharge disposition served as the reference categories for race/ethnicity and ED disposition, respectively. Odds ratios (OR) are presented with corresponding 95% confidence intervals (CI) and p-values. No statistically significant associations were observed. Asterisks indicate statistical significance: * p < 0.05, ** p < 0.01, *** p < 0.001 -: Intentionally left blank

ACS diagnosed in ED	OR	95% CI	p-value
A typical symptom presentation	0.90	0.32-2.49	0.83
Age	1.04	0.98-1.11	0.22
Male	1.20	0.43-3.34	0.73
Non-Hispanic Black	1.17	0.26-5.37	0.84
Hispanic	1.14	0.15-0.84	0.90
Return/Transfer to nursing home	0.71	0.15-3.36	0.66
Transfer to another facility (not usual)	1.96	0.53-7.27	0.31

From Table 2 above, it is evident that atypical symptom presentation was not significantly associated with ACS diagnosis in the ED (OR: 0.90, 95% CI: 0.32-2.49, p = 0.83). Increasing age showed a modest but non-significant trend toward higher odds of ACS diagnosis (OR: 1.04, 95% CI: 0.98-1.11, p = 0.22). Further, male sex was not associated with increased odds of ACS diagnosis (OR: 1.20, 95% CI: 0.43-3.34, p = 0.73). Compared to non-Hispanic White patients (reference group), odds of ACS diagnosis did not differ significantly among non-Hispanic Black patients (OR: 1.17, 95% CI: 0.26-5.37, p = 0.84) or Hispanic patients (OR: 1.14, 95% CI: 0.15-0.84, p = 0.90). No ACS cases were recorded in the non-Hispanic Other group, so it was excluded from the model. Consequently, regarding ED disposition, patients who were transferred to another facility had higher, but non-significant odds of ACS diagnosis (OR: 1.96, 95% CI: 0.53-7.27, p = 0.31), while those discharged to nursing homes showed no meaningful association (OR: 0.71, 95% CI: 0.15-3.36, p = 0.66). Importantly, the variable "Admitted to hospital from ED" was excluded from the final model due to perfect collinearity, as all patients diagnosed with ACS were admitted, leaving no variation to analyze statistically.

## Discussion

This retrospective analysis aimed to investigate whether atypical symptom presentations in older adults were associated with a reduced likelihood of Acute Coronary Syndrome (ACS) diagnosis during emergency department (ED) visits. Using nationally representative data from the National Hospital Ambulatory Medical Care Survey (NHAMCS) spanning 2014 to 2020, we analyzed 2,470 ED visits by adults aged 65 years and older. Among these, only 15 cases (0.6%) were diagnosed with ACS in the ED. Despite clinical concern that atypical symptoms may lead to missed diagnoses, our analysis did not find a statistically significant association between atypical symptom presentation and ACS diagnosis.

In this study, nearly half of all older adults presenting with ACS (7; 46.7%) exhibited atypical symptoms such as general weakness, shortness of breath, dizziness, syncope, or abdominal discomfort rather than classic chest pain. However, atypical presentation was not associated with decreased odds of ACS recognition in the ED (OR: 0.90; 95% CI: 0.32-2.49; p = 0.83). These findings suggest that, within this nationally sampled dataset, the presence of atypical symptoms did not independently predict whether ACS would be diagnosed during the ED encounter. The multivariable analysis also revealed no significant associations between ACS diagnosis and age, sex, race/ethnicity, or final ED disposition. Though age showed a small, non-significant trend toward increased ACS odds (OR: 1.04), this did not reach statistical significance. Similarly, patients transferred to other facilities or nursing homes did not exhibit significantly different odds of ACS diagnosis compared to those discharged home. "Admitted to hospital from ED" was excluded from the final model due to perfect collinearity, as all patients diagnosed with ACS were admitted.

These findings align with prior research indicating that atypical symptoms are frequently observed among older adults with Acute Coronary Syndrome (ACS), yet do not always result in diagnostic delays, particularly in emergency departments with robust triage systems and established cardiac workup protocols. Older patients often present without classic chest pain, instead reporting symptoms such as dyspnea, fatigue, nausea, or syncope, which can complicate early recognition of ACS. In a review by Engberding and Wenger [23], the authors highlight that atypical presentations are common in elderly populations and are frequently accompanied by non-specific or inconclusive electrocardiographic findings, contributing to the complexity of diagnosis. However, unlike hospital-based studies that utilize detailed clinical records and laboratory data, the NHAMCS dataset lacks key diagnostic information such as ECG interpretation, troponin levels, and clinical notes, limiting the ability to assess the diagnostic accuracy or timeliness of ACS recognition in this study setting [23].

An important strength of our study lies in its use of nationally representative data, which allows for generalizability across a broad spectrum of ED settings in the United States. Furthermore, our strict focus on older adults, a population at particularly high risk for both atypical presentations and adverse cardiac outcomes, provides clinically relevant insight into real-world ED evaluation practices.

Limitations

This study has several limitations that may have impacted the interpretation of results. Most notably, the NHAMCS dataset lacks critical clinical details such as electrocardiogram (ECG) findings, troponin levels, and other cardiac biomarkers, which are essential for confirming Acute Coronary Syndrome (ACS). As a result, diagnostic accuracy could not be directly assessed, and proxy variables, namely ICD-9-CM diagnosis codes and Reason for Visit (RFV) codes, were used to infer ACS diagnoses and symptom types. These proxies, while structured, may not fully capture clinical nuance or provider judgment, and are susceptible to misclassification. Additionally, the regression analysis was further limited by the exclusion of the variable "admitted to hospital from ED" due to perfect collinearity, as all ACS-diagnosed patients were admitted. This removed a potentially meaningful predictor from the model. Additionally, the low number of ACS cases (n = 15) among over 2,400 ED visits significantly limited statistical power and the ability to detect meaningful associations. Also, as with many secondary datasets, NHAMCS is not designed for rare-event analysis or long-term follow-up. We could not track patients beyond the ED visit, which prevented identification of delayed or missed ACS diagnoses that may have occurred during hospitalization or post-discharge. Lastly, the dataset lacks several clinical covariates, including risk scores, provider notes, and pre-hospital care data, leaving the potential for residual confounding. Therefore, it is recommended that future studies should prioritize access to richer clinical datasets that include objective diagnostic measures (e.g., ECGs, troponin levels), provider documentation, and follow-up outcomes. Linking ED data to inpatient records or using prospective cohort designs could improve detection of delayed or missed ACS cases and offer a more comprehensive understanding of diagnostic pathways, particularly in high-risk older adults presenting with atypical symptoms.

## Conclusions

In this nationally representative analysis of emergency department visits by older adults, nearly half of the patients diagnosed with Acute Coronary Syndrome (ACS) presented with atypical symptoms. However, atypical presentation was not significantly associated with reduced odds of ACS diagnosis in the ED. Although limited by the low number of ACS cases and lack of detailed clinical data, this study reinforces the ongoing challenge of recognizing ACS in older adults who do not present with classic chest pain. Continued efforts to improve early identification, especially through enhanced clinical data collection and integrated diagnostic tools, remain essential for reducing missed or delayed ACS diagnoses in this vulnerable population.
